# Immunocompetent murine models for the study of glioblastoma immunotherapy

**DOI:** 10.1186/1479-5876-12-107

**Published:** 2014-04-29

**Authors:** Taemin Oh, Shayan Fakurnejad, Eli T Sayegh, Aaron J Clark, Michael E Ivan, Matthew Z Sun, Michael Safaee, Orin Bloch, Charles D James, Andrew T Parsa

**Affiliations:** 1 Department of Neurological Surgery, Northwestern University, Feinberg School of Medicine, 676 N. St. Clair St., Suite 2210, Chicago, IL 60611-2922, USA; 2 Department of Neurological Surgery, University of California, San Francisco, 505 Parnassus Ave., Room 779M, San Francisco, CA 94143-0112, USA

**Keywords:** Glioblastoma, Astrocytoma, Glioma, Immunotherapy, Preclinical, Animal models, Murine, Immune response, Immunosuppression

## Abstract

Glioblastoma remains a lethal diagnosis with a 5-year survival rate of less than 10%. (NEJM 352:987-96, 2005) Although immunotherapy-based approaches are capable of inducing detectable immune responses against tumor-specific antigens, improvements in clinical outcomes are modest, in no small part due to tumor-induced immunosuppressive mechanisms that promote immune escape and immuno-resistance. Immunotherapeutic strategies aimed at bolstering the immune response while neutralizing immunosuppression will play a critical role in improving treatment outcomes for glioblastoma patients. *In vivo* murine models of glioma provide an invaluable resource to achieving that end, and their use is an essential part of the preclinical workup for novel therapeutics that need to be tested in animal models prior to testing experimental therapies in patients. In this article, we review five contemporary immunocompetent mouse models, GL261 (C57BL/6), GL26 (C57BL/6) CT-2A (C57BL/6), SMA-560 (VM/Dk), and 4C8 (B6D2F1), each of which offer a suitable platform for testing novel immunotherapeutic approaches.

## Introduction

With a median survival of just 15months, glioblastoma (GBM) is a notoriously lethal tumor of the central nervous system marked by significant treatment resistance [1]. Standard treatment for patients with GBM is maximum safe resection of tumor followed by radiation with concurrent temozolomide [1]. Glioma immunotherapy, a promising alternative to such aggressive cytotoxic adjuvants, is a highly specific, minimally toxic modality capable of killing tumor cells while sparing normal tissue [2-5]. Immunotherapy in glioma can also activate immune surveillance, and thereby offers the potential for long-term control of this lethal disease [2-6]. However, tumor-induced immunosuppression exists as a considerable barrier to achieving successful immunotherapeutic treatment of GBM and other tumors. GBMs inhibit immune function, systemically as well as within the tumor microenvironment, causing many patients to present with impaired cell-mediated immunity [3,5,7]. Implicated mediators of immunosuppression include regulatory T cells (T_regs_), myeloid-derived suppressor cells, and M2 macrophages [8]. Tumor heterogeneity [9] and immune escape mechanisms [10] further complicate immunotherapeutic treatment efficacy.

Focused research strategies designed to mitigate immunosuppressive mechanisms could contribute information of critical importance to improving GBM patient outcomes. Preclinical research using immunocompetent mouse models offers a means for studying important interactions between glioma, the immune response, and therapeutics, and for hypothesis-driven evaluation of novel approaches for treating GBM [11]. Here, we present a review on immunocompetent murine glioma models and examine their applications for glioma research, placing specific emphasis on their value to the field of glioma immunotherapy.

## Murine models for glioma - an overview

A useful experimental glioma model should have the following features: 1) *in vitro* sustainability, 2) *in vivo* recapitulation of glioma features (e.g. invasion and angiogenesis), 3) amenability to genetic manipulation, 4) facile transplantation, 5) reproducible and predictable growth characteristics [12,13]. Histopathology, tumor biology, molecular profiles, and invasiveness are all important characteristics to preserve in order to replicate glioma behavior *in vivo*.

Two major considerations are important when assessing preclinical models for GBM, the first of which is generation of the tumor itself and the stable transplantable cell lines that follow. GBM tumors can be generated spontaneously, or through induction using chemicals or viruses [14]. Spontaneous tumors, albeit rare and difficult to cultivate without a large host of animals, perhaps best reflect the natural course of human GBM [12,14]. However, for the purpose of increasing expediency and efficiency of time plus effort, tumor induction methods have been more commonly used. Seligman and Shear described the first successful experimental brain tumor in 1939 through intracranial (IC) implantation of 20-methylcholanthrene, leading to the development of gliomas and meningeal fibrosarcomas [15]. Viral induction with DNA or RNA viruses has also been widely used for inducing rodent model GBM and has made significant headway over the past few decades [16-19]. However, this approach presents many challenges, among which include potential harm to laboratory personnel, high maintenance requirements, and incomplete tumor penetrance [16-18].

The second consideration is the method of transplantation. Transplantation methods for murine models of glioma can be classified in three ways: 1) implantation of syngeneic primary tumor cells or cultured cell lines into immunocompetent hosts, 2) implantation of human glioma cells into immunocompromised mice (xenografts), or 3) implantation of cells that have been subjected to manipulation of oncogenes or tumor suppressor genes [12,20]. Subcutaneous syngeneic transplantation of tumor cells has a long history, initially having been demonstrated over 70years ago [21]. The first report of using athymic (nude) mice as hosts for human tumor cell propagation [22] was followed by the demonstration of human GBM xenograft establishment by Rana *et al*. [23]. Danks and colleagues described the first transgenic astrocytoma model in 1995 by inducing astrocyte expression of the pro-tumorigenic SV40 large T antigen [24].

Knowledge of model advantages and disadvantages is critical for selecting the best-fit model for achieving study objectives. While there is no single murine model that is appropriate for all types of preclinical research, spontaneous tumors in syngeneic, immunocompetent mouse models provide the most versatile tool for studying immunotherapy and immunosuppression in GBM. Comparatively, xenografts can be modified for the purpose of evaluating GBM immunological treatment modalities, [25-27] but their use is somewhat limited due to a compromised host immune system [20,28,29]. Transgenic models that have been engineered to spontaneously develop GBM in immunocompetent hosts have seen increasing use in glioma immunotherapy studies, although these models, in comparison to transplantation models, suffer from reproducibility, latency of tumor formation, and cost [28,29]. Transgenics may also result in mixed tumors of diverse histological grades, such that many fail to recapitulate key features of GBM [29,30]. Moreover, their genetic alterations can interfere with host immune functions such as lymphopoiesis and clonal expansion, which are important pathways to conserve for immunotherapy research in glioma [20].

In that manner, several syngeneic immunocompetent models are available for preclinical utilization. In the following sections, a brief overview and applications in GBM immunotherapy for each syngeneic model is presented. It should be noted that while each of these tumors arose spontaneously at the time of their discovery, they have since been maintained and experimentally utilized through serial transplantation or generation of transplantable cell lines.

## SMA-560-VM/Dk model

### Origins and tumor characteristics

In 1971, H. Fraser described the first incidence of a spontaneous glioma within the VM mouse strain [31]. Initially, these tumors, which resembled anaplastic astrocytomas, were restricted to *in vivo* studies only, as tumorigenicity was lost with repeated *in vitro* passaging of tumor explant cultures [31]. In 1980, Serano and colleagues successfully established five tumor cell lines (P492, P496, P497, P540, P560) following serial transplantation of a spontaneous murine astrocytoma. Cell lines were developed via homogenization of tumor tissue, *in vitro* culturing, and transplantation into VM/Dk mice. Although the P492, P496, and P497 cell lines demonstrated variable tumorigenicity after serial passaging, P540 and P560 maintained tumorigenicity throughout all passages [13]. Median survival for animals bearing SMA-560 tumors has been reported to be approximately 26 days when implanted intracranially with 1×10^4^ tumor cells/5 μl [32].

SMA-560 tumor cells provide excellent representation of anaplastic astrocytoma, with low S-100 expression and high expression of glial fibrillary acid protein (GFAP) and glutamine synthetase, thus supporting the astrocytic lineage of derivative tumors [13,33,34]. These tumors lack Class II but do express Class I Major Histocompatibility Complex (MHC) at low levels, which highlights their potential for antigenic recognition by traditional effector T cells [35]. Of interest, brain tumors derived from SMA-560 cells express transforming growth factor β (TGF-β), [35] an immunosuppressive protein known to be secreted by GBM [36]. TGF-β expression lends great value to this model, although it has failed to experience widespread use, likely due to its lack of commercial availability, thereby restricting its use to a few academic centers.

### Applications in immunotherapy research for GBM

The SMA-560 model has experienced occasional use, such as in the study by Sampson *et al*., the results of which showed that manipulation of SMA-560 cells for hyper-secretion of select cytokines, specifically interleukin (IL)-2, IL-4, or tumor necrosis factor α, resulted in an increase in median survival of VM/Dk mice following IC injection of modified cells (p < 0.0001). SMA-560 cell modification for increased production of IL-3, IL-6, interferon-γ, Cluster of Differentiation (CD)80, or granulocyte-macrophage colony-stimulating factor, had no significant effect on host survival [35].

Recently, Miller *et al*. showed that SMA-560 cells transfected to over-express a soluble form of the CD70 ligand reduced tumor growth rate and increased host animal survival. In addition, several long-term survivors from the group injected with CD70-modified cells demonstrated resistance to tumor re-challenge. Finally, the results of this study revealed that improved animal subject outcomes were contingent upon activation of a robust cytotoxic immune response. Immunohistochemical analysis of tumor samples revealed that mice with tumor cells expressing soluble CD70 had greater infiltration of CD_8_^+^ T cells in the tumor periphery, and that depletion of CD_8_^+^ T cells reversed the benefits of soluble CD70 to overall survival [37].

The SMA-560-VM/Dk model has also been used to investigate tumor vaccines. Heimberger *et al*. pulsed bone marrow-derived dendritic cells (DCs) with SMA-560 homogenate, and administered pulsed DCs to VM/Dk mice prior to IC implantation of SMA-560 cells. Compared to control, mice treated with pulsed DCs demonstrated median survival of >65 days versus 25 days, thus representing a 160% increase in median survival (p = 0.016). When surviving immunized mice were re-challenged with tumor 50 days following initial treatment, increased survival again resulted (>50 days), thereby providing evidence for the acquisition of long-lasting anti-tumor immunity. Both cell-mediated and humoral immunity were found to be involved in the generation of this potent therapeutic response [32]. More recently, Sampson *et al*. utilized the SMA-560 model to test the efficacy of genetically modified T cells, which were modified to express chimeric antigen receptors targeting the epidermal growth factor receptor vIII (EGFRvIII), a known glioma antigen. When this was administered to tumor-bearing mice, growth of SMA-560 tumors was abrogated, and treatment conferred resistance to tumor re-challenge [38].

## CT-2A; C57BL/6 model

### Origin and tumor characteristics

Developed specifically for characterizing ganglioside distribution in murine neural tumors, the CT-2A cell line was established by Seyfried *et al*. in 1992 through chemical induction with 20-methylcholanthrene. Following serial transplantation of tumor fragments into C57BL/6 mice, this syngeneic model for highly malignant, poorly differentiated anaplastic astrocytoma resulted in 100% mortality within 3–8 weeks [39]. In 2007, Martinez-Murrillo *et al.* standardized techniques for establishing CT-2A tumors from cultured CT-2A cells, as opposed to solid tumors, and demonstrated a survival range of 15–20 days with IC injections of 8×10^4^ tumor cells/4 μl [40].

Histologically, CT-2A tumors manifest features of high-grade astrocytomas including pleomorphism and high cellular density, but can undergo malignant transformation with evidence of pseudopalisading necrosis [40]. Tumors are angiogenic, occasionally cystic, and infiltrative, with tumorigenesis rates reported up to 100% [40-43]. Compared to established glioma cell lines, CT-2A cells are significantly more proliferative and invasive (p < 0.05), [41] but less invasive than other mouse brain tumors [44]. Overall, the CT-2A model is considered to accurately represent several GBM characteristics including intra-tumoral heterogeneity, *in vivo* migratory patterns, radio-resistance, and chemo-resistance [40].

As recently elucidated by Binello *et al*., CT-2A tumors also share similarities with neural stem cells, as they form neurospheres when cultured in serum-free media, much like primary human GBMs grown *ex vivo*, [45] and express stem cell markers such as CD133, Oct, and nestin [41]. Cells expressing Sox9 and Sox10 localize to the periphery of CT-2A tumors [40]. Similarities between CT-2A tumor cells and brain tumor stem cells (BTSCs), which are capable of self-renewal, express CD133, and possess profound tumor-forming capacity, [46-48] may account for their high tumorigenic potential [44]. Phenotypically, the “stemness” of CT-2A tumors manifests as significantly enhanced proliferative and invasive capacity *in vitro*[41]. Importantly for glioma immunotherapy, however, the consequences of neurosphere formation and culture on CT-2A tumor immunogenicity is currently unknown and requires further investigation.

### Applications in immunotherapy research for GBM

By virtue of its BTSC-like properties, the CT-2A model could provide a resource for studying tumor stem cells in an immunocompetent environment. Due to mounting evidence that BTSCs negatively impact overall and progression-free survival, [49] while contributing to treatment resistance in high-grade gliomas, [50,51] therapeutic targeting of BTSCs is a subject of some importance. BTSCs induce immunosuppression by expressing Programmed Death Ligand-1 (PD-L1) and TGF-β1, as well as by inhibiting T cell proliferation, inducing T cell apoptosis, and enhancing T_reg_ function [52,53]. While to date, BTSC-focused GBM preclinical research has not been especially active with respect to the development of immunotherapeutic strategies, several groups have recently demonstrated the potential of targeting BTSCs using immunotherapeutic approaches. Morgan *et al*. tested the efficacy of T cells genetically engineered to target EGFRvIII on glioma stem cells and found that these lymphocytes induced significant antitumor effects [54]. Brown *et al*. also found that cytotoxic T lymphocytes (CTLs) engineered to target the IL13Rα2 receptor, which can be mutated in GBM, were able to induce tumor regression in xenografts established from stem cells [55]. In non-GBM cancers, passive antibody-mediated approaches and antibody-drug conjugates directed against cancer stem cells have been shown to reduce disease burden [56-59]. Theoretically, BTSCs can be targeted through vaccine therapies, although a potential challenge to this approach concerns BTSC avoidance of immune surveillance [60]. A key research aim, for which the CT-2A model may be well-suited, involves determining how to augment the immunogenicity of CD133^+^ BTSCs, including the identification of novel epitopes to target [60].

CT-2A tumors are also deficient in the phosphatase and tensin homolog (PTEN) protein, leading to dysregulation of the phosphatidylinositol-3 kinase (PI3K) pathway [61]. PTEN mutations also carry clinical significance, and are observed in 40% of high-grade human gliomas as well as 70% of glioma cell lines [61,62]. PTEN mutations contribute to tumor-induced immunosuppression, and thus the CT-2A model can be utilized to devise strategies for mitigating PTEN deficiency-associated immune effects [63].

## GL261; C57BL/6 model

### Origin and tumor characteristics

The Glioma 261 (GL261) orthotopic model for murine glioma was established in 1970 via chemical induction with methylcholanthrene. Ausman *et al*. transplanted tumor fragments subcutaneously and intracranially into C57BL/6 mice, with the latter resulting in a median survival of 24–25 days when implanted with 1×10^5^ tumor cells/10 μl [64,65]. Stable GL261 cell lines for transplantation were constituted in the mid-1990s [65]. Although the GL261 tumors most resemble ependymoblastomas on histology, GL261 tumors closely mimic GBM phenotypes [64]. They stain positively for the GBM marker vimentin [66] and harbor activating mutations of the K-ras oncogene as well as mutations of the p53 tumor suppressor gene, resulting in high expression of c-myc [65]. Similar genetic derangements have been reported in human gliomas [67-69]. GL261 tumors are partially immunogenic, as they express high levels of MHC I. However, GL261 expression of MHC II, B7-1, and B7-2, the latter two of which are co-stimulatory molecules required for T cell activation, is limited [65]. Reduced MHC and B7 expression is also characteristic of GBM cell lines and CD133^+^ BTSCs, which contributes to their escape from immune surveillance [60,70,71].

Tumors established from GL261 cells recapitulate many characteristics of GBM. Tumor formation proceeds through four stages, over a four-week period, following implantation: perivascular organization, proliferation near vasculature, hypoxia through blood vessel degeneration, and neovascularization towards necrotic regions [72]. Histologic analysis reveals pleomorphism, pseudopalisading necrosis, and angiogenesis [72]. While invasive, GL261 tumors are not known to be metastatic [65] and, importantly, these tumors do not spontaneously regress as other murine tumors are known to do on occasion [73].

### Applications in immunotherapy research for GBM

The GL261 murine model has perhaps been the most extensively used for preclinical testing of immunotherapeutic approaches for GBM [74]. Among these studies are: the use of adoptive T cell transfer to restore and induce long-term immunity [75]; the use of antibodies to improve antitumor T cell activity via augmentation of costimulatory signaling [76]; and the abrogation of the survival advantage of T_regs_[77]. Gene therapy studies, involving tumor modification for production of inflammatory cytokines (e.g. IL-2) to enhance tumor immunogenicity [78] as well as with IL-12-expressing DNA plasmids to slow tumor growth and stimulate a robust CTL response, [79] have also utilized the GL261 model.

The GL261 model has also been widely used in support of vaccine-based studies. GL261 cells express unique tumor antigens, including HMP/AN2, [80] EphA-2, [81] and GARC-1, [82] and these induce a GL261-specific CTL response. GL261 vaccines, used for pulsing DCs, have been curative and, at times, even preventative of tumor engraftment [83-85]. DC vaccines have also been augmented using adjuvants such as plasmid vectors for IFN-γ-inducible protein-10 (IP-10) [86] or by antibody-mediated depletion of T_regs._[87] The results of these studies have helped validate GL261 as the model of choice when investigating immunotherapeutic treatment modalities.

The GL261 model has also been used to test experimental methods for mitigating GBM-induced immunosuppression. For instance, in a study by Ueda *et al*., mice were treated with peptide vaccinations using GL 261-specific antigens and a TGF-β neutralizing antibody (1D11). Mice receiving both treatments showed 60% 100-day survival, in contrast to the 0-20% survival rates for mice receiving treatments independently. Analysis of animal subjects from this study revealed significantly elevated CTL activity within the lymph nodes and spleens, as well as greater immune cell infiltration, with concurrent reduction of T_regs,_ in the brains of the mouse hosts. Overall, treatment was associated with promoting the Th_1_ phenotype [88].

The GL261 model has also been used for studying the immunosuppressive effects of TGF-β, which promotes T_reg_ activity, on B and T cell function [36,89,90]. Additional inhibitory mechanisms beyond TGF-β can be studied using this model. Given GL261 deficiency in PTEN, GL261 tumors accurately model PI3K pathway dysregulation, which is known to promote glial tumor development [91]. Importantly, PTEN mutations up-regulate expression of PD-L1, a cell surface protein that can be expressed in GBM tumors but not in normal physiologic states [10]. PD-L1 promotes immunosuppression by inducing T lymphocyte apoptosis, [92] and monocytes exposed to PD-L1^+^ gliomas adopt similar expression patterns of PD-L1, leading to increased T cell apoptosis and tumor resistance to immunotherapy [93,94]. Devising methods to target and reverse PD-L1-mediated immunosuppression is thus an important objective for optimizing immunotherapies [95].

Since GL261 cells express stem cell markers such as CD133 when grown in serum-free media, these cell subpopulations can be isolated and propagated for experimental use. Importantly, when grown in serum-positive media, GL261 cells differentiate and do not express CD133, [96] as has been similarly reported with primary human GBM cell lines [45]. Isolation of CD133^+^ GL261 cells appears to increase tumorigenicity, as even IC implantation of CD133^+^ GL261 cells at small volumes (~100 cells) leads to tumor formation and GL261 neurosphere formation is also greater when culturing CD133^+^ cells in serum-free media [96]. Thus, as is the case for the CT-2A model, GL261 tumors may find use for studying BTSCs and immunosuppression. However, one potentially important consideration to this is that GL261 stem cells appear to enhance tumor immunogenicity. As demonstrated by Pellegatta *et al*., DCs pulsed with tumor lysates from GL261 neurospheres, as opposed to normal GL261 cells, generated a more robust T cell and antitumor immune response [97]. More recently, Xu *et al*. lent further evidence to this by showing that immunotherapy with DCs pulsed with GL261 stem cell lysates or DCs pulsed with GL261 lysates were able to prevent tumor formation in 37.5% and 0% of mice, respectively, and induced a significant CTL response [98].

Despite the extensive information yield from using the GL261 cells, an inherent disadvantage of this model is its moderate immunogenicity which may complicate interpretation of experimental data [65]. Reduced immunogenicity can confound evaluation of responses to immunotherapy, and extrapolation of results to the clinical setting, as GBM is known to be an immune-privileged tumor that evades host immune recognition [99]. Nevertheless, the GL261 system presents one of the best characterized syngeneic, immunocompetent models, and it is likely that this model will continue to see extensive use in immunotherapy preclinical research.

## GL26; C57BL/6 model

### Origin and tumor characteristics

The less commonly utilized GL26 glioma cell line closely resembles GL261, and was first chemically induced by Sugiura in 1969 [64]. Much like its GL261 analog, GL26 tumors bear greatest histologic resemblance to ependymoblastomas. However, they differ slightly in that GL26 tumors tend to demonstrate greater necrosis and vascularity in addition to being more hemorrhagic [64]. Overall, GL26 gliomas possess characteristic GBM features that lend well to GBM research: they stain positive for vimentin and exhibit cellular pleomorphism, hypercellularity, nuclear atypical, and inflammation [66]. Although GL26 cells express Class I MHC antigens, Class II MHC antigens are undetectable [100]. Median survival following IC tumor implantation of 2×10^4^ tumor cells/0.5 μl is 31 days [66].

### Applications in immunotherapy for GBM

The GL26; C57BL/6 model has been utilized to study several immunotherapeutic approaches and, while it has not seen as extensive use as GL261, there is evidence to suggest that this model can prove invaluable to the study of immunotherapy. GL26 tumors express melanoma-associated antigens (MAAs) “gp100” and “tyrosinase-related protein 2 (TRP-2),” both of which can be immunogenically targeted for CTL-mediated destruction. Prins *et al*., for example, employed this strategy to great effect by vaccinating mice with MAA-pulsed DCs, which led to a robust antitumor immune response and significantly prolonged survival [101]. Alternatively, Kim and colleagues have shown similar antitumor efficacy with the administration of genetically engineered IL-12-expressing DCs pulsed with GL26 tumor lysates [102].

Other strategies tested with this model include T_reg_ depletion using PC61, and antibody directed against CD25, which is one of the primary markers for T_regs_. Curtin *et al*. found that, although PC61 was unable to induce immunologic memory against tumors and prevented the expansion of tumor-specific T lymphocytes, it did inhibit tumor growth, dramatically reduce tumor infiltration with T_regs_, and prolong overall survival in the context of low tumor burden [103]. The benefits of combinatorial chemo-immunotherapy consisting of pulsed DCs and temozolomide have also been investigated. When Park *et al*. treated tumor-bearing mice with low-dose temozolomide and DCs transfected for survivin, an anti-apoptotic highly expressed in gliomas, prolonged survival was seen and this was due to increased cross-priming of tumor-specific T cells [104]. Another group similarly utilized low-dose temozolomide and DCs pulsed with tumor lysates to show increased cross-priming, immune infiltration, and survival, thereby highlighting the potential promise for this therapeutic approach [105].

## 4C8; B6D2F1 model

### Origin and tumor characteristics

Weiner *et al*. developed the 4C8-B6D2F1 model to address shortcomings observed with other murine glial tumors [106]. The 4C8 tumor was established from clonal cell lines of a glial tumor known as MOCH-1, derived from a transgenic mouse. This approach should, in fact, be generalizable to the development of dozens of syngeneic, immunocompetent engraftment models, using tumor cells from the many brain tumor transgenic mouse models that have been created over the past 2 decades of genetically engineered mouse model research. In contrast to MOCH-1, which strongly resembles GBM, 4C8 cells adopt oligodendrocytic characteristics *in vitro* but convert to GFAP^+^ astrocytes when exposed to serum [107]. IC implantation into B6D2F1 mice produces pleomorphic, highly cellular tumors, with extensive invasion into ventricles and meninges [106]. B6D2F1 tumors also express components of MHC I and II molecules [108]. Overall, mice bearing B6D2F1 tumors have demonstrated a mean survival of approximately 51 days when intracranially injected with 1×10^6^ cells/5 μl [106].

### Applications in immunotherapy research for GBM

One application of this model has been for the analysis of effects from treating tumors with cationic liposomal non-coding plasmid DNA complexes (EV-CLDC), which demonstrated inhibition of tumor growth (p < 0.0001) [108]. In addition, intratumoral injections of vaccines with herpes simplex viruses, engineered to secrete IL-12, have been shown to promote significant anti-tumor activity, with immune cell infiltration, and minimal toxicity [109,110]. As a relatively new model, however, additional study is needed to reveal the full range of this model’s applications.

## Summary and conclusion

Animal models have been indispensable for the study of gliomagenesis, glioma progression, and experimental therapies. Transplant models offer the convenience of predictable tumor location and growth rate while facilitating the study of interactions between gliomas and the host immune system [111]. However, it is important to note that all animal models have certain deficiencies that place limitations on their use, [20] and knowledge of these, as well as model strengths, is essential for obtaining preclinical results that are meaningful for clinical translation [20].

The five syngeneic immunocompetent murine models reviewed, as summarized in Table 1, recapitulate certain histologic and biological characteristics of human astrocytomas and GBM, and their use of immunocompetent, syngeneic hosts make them well-suited for studying glioma immunology and a range of experimental immunotherapies. Preclinical findings from these murine models have already been translated to clinical trials in human glioma patients. For example, immunotherapeutic treatments utilizing DC vaccines pulsed with whole tumor homogenate [112] or tumor-specific peptides, such as for EGFRvIII, [113-115] owe their translational origins to successful studies using the GL261 model [84,116-119]. Other clinical trials for targets such as TGF-β [120,121] and gene therapeutic approaches [122] also have origins in preclinical studies using the SMA-560 [123] and GL261 models, [124,125] respectively.

**Table 1:** Immuno-competent syngeneic murine models of glioma

**Cell line**	**Host**	**Induction**	**Histology**	**Specific and potential applications in immunotherapy**	**Refs**
P560	VM/Dk	Spontaneous	Anaplastic Astrocytoma	• Vaccine studies (e.g. DC)	13, 31-38
				• Gene therapy studies (e.g. IL-2, CD70)	
				• Reversal of immunosuppression in glioma (e.g. TGF-β)	
CT-2A	C57BL/6	Chemical	Anaplastic Astrocytoma	• Tumor stem cells (BTSCs)	39-63
				• Reversal of immunosuppression in glioma (e.g. TGF-β, PTEN)	
GL261	C57BL/6	Chemical	GBM/Ependymoblastoma	• Tumor stem cells (BTSCs)	10, 35, 60, 64-99
				• Vaccine studies (e.g. dendritic cells)	
				• Gene therapy studies (e.g. IL-2)	
				• Adoptive T cell, antibody, and T_reg_ depletion studies	
				• Reversal of immunosuppression in glioma (e.g. TGF-β, PTEN)	
GL26	C57BL/6	Chemical	GBM/Ependymoblastoma	• Vaccine studies (e.g. dendritic cells)	64, 66, 100-105
				• Gene therapy studies (e.g. IL-12)	
				• T_reg_ depletion studies	
				• Chemo-immunotherapy	
4C8	B6D2F1	Transgenic	Oligodendroglioma, Astrocytoma	• Vaccine studies (e.g. HSV)	106-110
				• Gene therapy studies (e.g. plasmids)	

Refs = References; DC = Dendritic Cells; IL-2 = Interleukin-2; IL-12 = Interleukin-12; CD70 = Cluster of Differentiation70; TGF-β = Transforming Growth Factor-β; BTSC = Brain Tumor Stem Cell; PTEN = Phosphatase and Tensin Homolog; GBM = Glioblastoma; T_reg_ = Regulatory T Cell; HSV = Herpes Simplex Virus.

Contemporary literature indicates that the GL261 model has been most frequently used. However, further research using SMA-560, CT-2A, GL26, and 4C8 tumors seems likely to reveal additional glioma immunotherapy applications for these models as well. Given the promise of immunotherapy as part of a multimodal treatment paradigm for GBM, such *in vivo* models will continue to prove invaluable in the future.

## Abbreviations

GBM: Glioblastoma; Tregs: Regulatory T cells; IC: Intracranial; MHC: Major histocompatibility Complex; TGF-β: Transforming growth factor β; IL: Interleukin; CD: Cluster of differentiation; DC: Dendritic cell; BTSC: Brain tumor stem cell; PD-L1: Programmed death ligand-1; PTEN: Phosphatase and tensin homolog; GL261: Glioma 261; EGFRvIII: Epidermal Growth Factor Receptor vIII; CTL: Cytotoxic T lymphocyte.

## Competing interests

The author(s) declare that they have no competing interests to report.

## Author’s information

OB is an Assistant Professor in the Department of Neurological Surgery at Northwestern University Feinberg School of Medicine, and the Reza and Georgianna Khatib Endowed Chair in Skull Base Tumor Surgery. CDJ is the Kathleen M. Plant Distinguished Professor in the Department of Neurological Surgery at University of California, San Francisco, the Berthold and Belle N. Guggenhime Endowed Chair, and the Associate Director and Principal Investigator at the Brain Tumor Research Center at University of California, San Francisco. ATP is a Michael J. Marchese Professor and Chair in the Department of Neurological Surgery at Northwestern University Feinberg School of Medicine.

## Authors’ contributions

TO participated in the design and coordination of the study, and wrote the manuscript. SF and ES helped draft the manuscript and collect relevant information. AC, MI, MZS, and MS were substantially involved in the design process, provided critical review of the manuscript, and assisted in the analysis and interpretation of the data. OB, CDJ, and ATP were instrumental in the coordination of the study and provided critical insight, direction, and revisions throughout the drafting process. ATP also spearheaded the study concept and design. All authors read and approved the final manuscript.
